# Supplementary material for: The causal relationship between the human gut microbiota and pyogenic arthritis: a Mendelian randomization study
Source: Front Cell Infect Microbiol. 2024 Nov 26;14:1452480. doi: 10.3389/fcimb.2024.1452480 (PMC11629706; doi:10.3389/fcimb.2024.1452480)
Supplement: Supplementary file 1 [file DataSheet1.zip › mendelian test/ebi-a-GCST90027715.csv_forest.pdf]

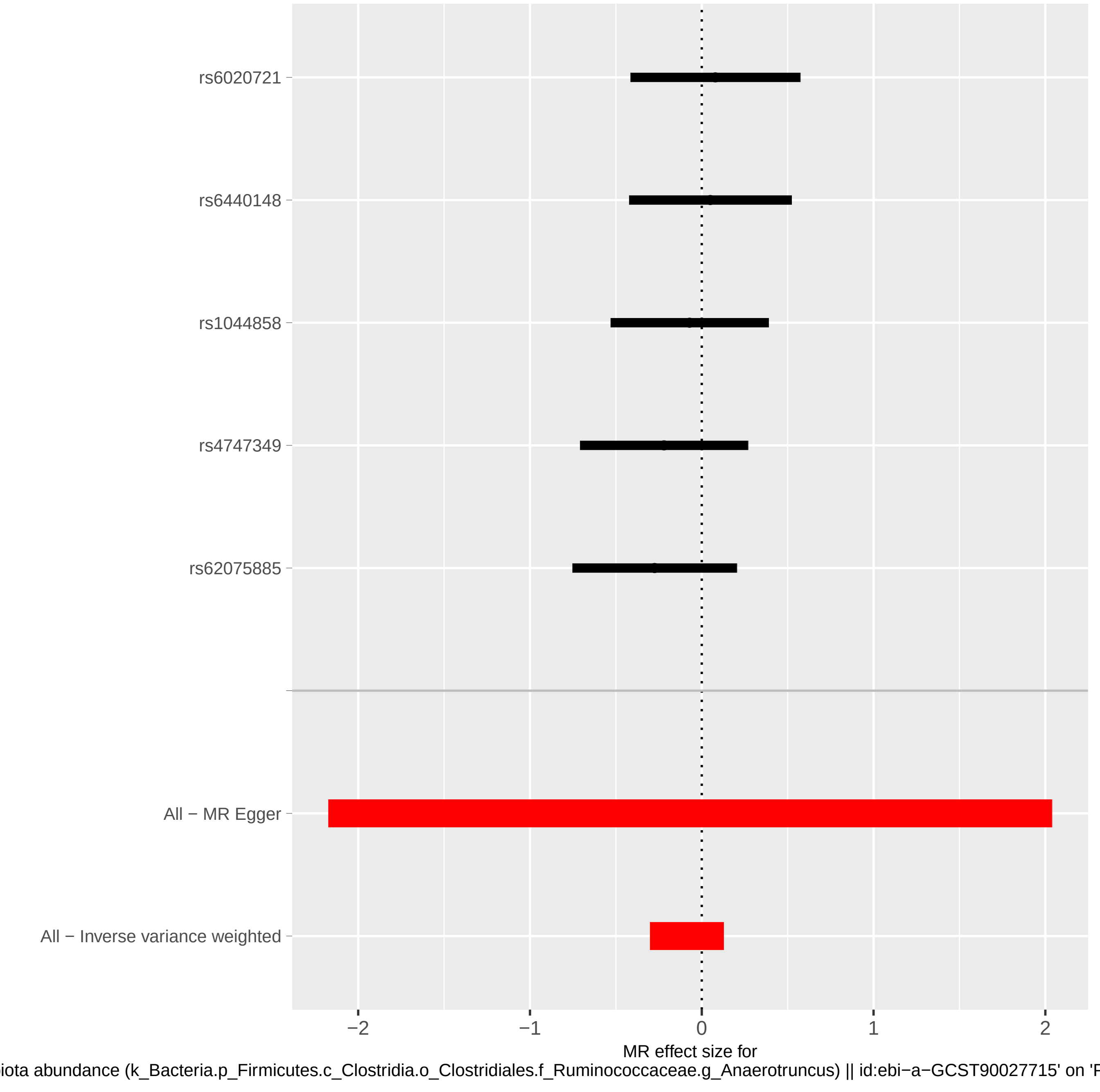

rs6020721
rs6440148
rs1044858
rs4747349
rs62075885
All − MR Egger
All − Inverse variance weighted
−2
−1
0
1
2
MR effect size for
iota abundance (k_Bacteria.p_Firmicutes.c_Clostridia.o_Clostridiales.f_Ruminococcaceae.g_Anaerotruncus) || id:ebi−a−GCST90027715' on 'F
